# Prehabilitation for cancer surgery: a systematic review of qualitative literature from experienced stakeholders

**DOI:** 10.1007/s00520-026-10811-x

**Published:** 2026-06-09

**Authors:** Rohan Miegel, David I. Watson, Raymond J. Chan, Matthew P. Wallen, Sarah C. Hunter

**Affiliations:** 1 Caring Futures Institute, College of Health and Enablement, Flinders University, Adelaide, South Australia Australia; 2 Division of Surgery and Perioperative Medicine, Flinders Medical Centre, Southern Adelaide Local Health Network, Adelaide, Australia; 3 College of Medicine and Public Health, Flinders University, Adelaide, South Australia Australia; 4 Department of Physiotherapy, Flinders Medical Centre, Southern Adelaide Local Health Network, Adelaide, Australia

**Keywords:** Cancer surgery, Prehabilitation, Systematic review, Barriers, Facilitators, Implementation

## Abstract

**Purpose:**

Multimodal prehabilitation for cancer surgery is increasingly being used in cancer care to improve patient outcomes. Despite the benefits of such programs on patient and health service metrics, many services and research trials report challenges with patient uptake and adherence as well as implementation into existing services. This systematic review sought to better understand the key enablers and barriers to participant uptake and adherence, to inform future service adoption recommendations.

**Methods:**

MEDLINE, CINAHL, Scopus and PsycInfo were systematically searched for qualitative and mixed-methods studies from January 2010 to July 2025. Two reviewers independently screened, extracted and conducted critical appraisal. A customised data extraction tool was developed, and study quality was critically appraised via the Mixed Methods Appraisal Tool (MMAT). Data were thematically synthesised into patient, clinician and system level factors influencing engagement.

**Results:**

Twenty-five studies were identified. Patient engagement factors included the importance of relationships (clinician-patient, family and patient peer groups), acknowledging individual circumstances with the need for individualised content, and the belief of the benefit of participation. Implementation factors included workforce capacity and capability, organisational system support, and utilising the preoperative window effectively. Combined factors were the delivery method and clinician awareness and attitudes towards prehabilitation.

**Conclusion:**

Engagement with prehabilitation is shaped by interconnected patient and system factors. Successful implementation requires addressing both individual patient needs and organisational infrastructure. Future research should expand beyond proving effectiveness to real-world integration.

**Implications for cancer survivors:**

Survivors benefit from tailored prehabilitation programs that consider personal circumstances and provide strong relational support. Embedding prehabilitation into standard care pathways promotes equitable access and empowers survivors to participate actively.

**Supplementary Information:**

The online version contains supplementary material available at 10.1007/s00520-026-10811-x.

## Introduction

Approximately 80% of all cancers require surgery at some point [1, 2]. There is global demand for cancer surgery, which is projected to increase by 52% from 2018 to 2040, with the majority of projected demand attributed to an increase in gastrointestinal and urological cancers [3]. Given this, there have been repeated and strong calls for preoperative optimisation of patients at risk of experiencing a complication, especially older, male, multiple comorbidities and those undergoing treatment with chemotherapy and/or radiotherapy before major surgery [4–6].

Undergoing major cancer surgery causes significant physiological, psychosocial stress [7, 8]. By supporting patients prior to surgery, multimodal prehabilitation (exercise, nutritional and psychological support) is a complex intervention that has been posited as a potential solution to mitigate the typical decline associated with cancer [8, 9]. There are now systematic reviews and several large clinical trials that demonstrate the effectiveness of prehabilitation as an intervention in multiple surgical populations—many of these oncological [10–13]. However, many of the trials describe issues with patient uptake and adherence. To illustrate this problem, a systematic review of 22 studies found up to 82% of patients decline engagement with a prehabilitation program [14].

To date, research investigating the uptake and adherence of prehabilitation has mostly been conducted through clinical trials, or rarely at sites with successful implementation, and has focused on interview- or survey-based methods. Frequent patient factors included were lack of information and awareness [15–20], location of services, overwhelm [15, 21] and competing interests and responsibilities [20, 22, 23]. Health professional factors included were busy workloads [24], short time frames [25], lack of staff and staff training [21, 26] and lack of trust in the intervention itself [19, 26, 27]. System barriers were infrequently reported, due to nature and intent of studies but did include lack of funding and administrative support and inadequate resources [19, 26, 28, 29].

These perspectives are seldom considered together, which does not acknowledge the interdependencies of engagement and implementation factors. For example, the field of implementation science outlines that successfully implementing evidence into practice requires understanding the complex interplay between evidence factors, individual factors and organisational factor and broader system factors [30]. Therefore, it is necessary to understand the factors at all levels of stakeholders and how they interact to provide recommendations. Without this, prehabilitation models of care may prove effective to improve care, but the format and delivery may not be acceptable to both patients and health service providers. This review seeks to add new knowledge by reviewing qualitative data from experienced patients, family members and clinicians to view patient engagement factors in the context of implementation.

The aim of this systematic review was therefore to extend the current literature base by answering: what are the barriers and facilitators to prehabilitation for cancer surgery from experienced patients, family members and clinicians in qualitative literature?

### Review objectives

1. To understand patient, family and clinician lived experience of prehabilitation for cancer surgery
2. To synthesise recent qualitative literature to understand the key factors to engagement in prehabilitation for cancer surgery

## Methods

In this systematic review, all original (quantitative, mixed-methods and qualitative) papers were included if they reported qualitative findings on enablers and barriers to prehabilitation. This review was reported in accordance with the Preferred Reporting Items for Systematic Reviews and Meta-Analyses (PRISMA) Guidelines and was registered with PROSPERO 2023 (ID number: CRD42023449414).

### Reflexivity statement

To ensure a balanced analysis, the research team comprised of both clinicians and researchers spanning surgery, nursing, physiotherapy, exercise physiology and psychology. The team has various experiences across prehabilitation, qualitative research and implementation science. In addition to having a broad and diverse team, specifically to mitigate any biased interpretation of results, reflexive practices were also utilised. This included independent screening, extraction and critical appraisal by multiple researchers with a validated tool, and frequent team discussion to refine interpretations and reach consensus. The aim was to improve the transparency and credibility of the analysis and synthesis.

### Search strategy

The initial search was conducted on 3rd August 2023. The search was developed and conducted in MEDLINE, CINAHL, Scopus and PsycInfo databases for articles published from January 2010 to August 2023. The search strategy, including all search terms as Electronic Supplementary Material, were reviewed by an academic librarian and translated into each database (see the Appendix). The search was updated in July 2025.

### Study eligibility

Studies that investigated attitudes and perceptions of prehabilitation services for cancer surgery were included. Patients and their families or carers were included to gain a wider consumer perspective towards engaging in prehabilitation services and health professionals were included to understand their attitudes towards the intervention, and to highlight enablers and barriers from a service provision perspective.

The included studies included the following outcomes: enablers or facilitators and barriers, attitudes and perceptions of prehabilitation services prior to their cancer surgery. Quantitative and mixed-method studies that reported enablers and barriers as secondary outcomes were included.

The definition of prehabilitation was kept broad purposefully. As many interventions to date have been unimodal, these were included as they are likely to have key insights that would be relevant despite the research question aiming for multimodal interventions. The time frame was decided due to prehabilitation programs and services being a recent and emerging field to include 13 years of literature.

Our inclusion/exclusion criteria are found in Table 1.

**Table 1:** Inclusion/exclusion criteria

Inclusion criteria	Exclusion criteria
Adults (> 18 years old) who had been diagnosed with any cancer type	Not cancer surgery, i.e. haematological, biopsy, reconstructions, etc.
Treatment had to include surgery (e.g. surgical resection)	Intervention was not pre-operative
Patients, family members and health professionals involved in the care of cancer patients	Intervention was not either exercise, nutrition or psychological intervention
Qualitative or mixed-/-multi-method evidence available in English	No intervention or only observational—just investigating, e.g. fear
Barriers, enablers, facilitators to participation in prehabilitation programs	Solely physical or psychological quantitative outcome measures
Prehabilitation—exercise and/or dietary/nutrition and/or psychological support	Majority Prehab naive participants
Study designs included interventional, quasi-experimental study designs that included randomised controlled trials and non-randomised controlled trials, cohort, observational studies (where prehabilitation services were embedded and not the main phenomenon), cross-sectional studies and feasibility studies	Study types excluded systematic reviews, umbrella reviews, book chapters, editorials, case studies, prospective studies, protocols, abstracts, theses, solely quantitative studies, abstracts and conference papers
English	Pre 2010

### Study selection

Selected studies were exported from the databases and then were imported into Covidence systematic review software where duplicates were removed. Two researchers (RM with RJ/SH/MW) screened titles, abstracts and full-text articles with the above criteria. Resolution of disagreement was discussed between two researchers to achieve consensus.

### Data extraction and appraisal

Data were imported into a data extraction template that was developed by SH, MW and RM in Microsoft Excel. RM extracted the data from selected studies. Extracted data included key study characteristics such as title, authors, country, aim, population, intervention, study design and key results.

Due the various study types, the Mixed Methods Appraisal Tool (MMAT) was used to critically evaluate the methodological quality of the included studies. The MMAT is frequently used for mixed-methods, quantitative and qualitative studies [31]. The MMAT assesses studies on several methodological criteria relevant to the study design. It does not provide an overall quality rating, rather the reported percentages indicate the proportion of criteria met by each study related to its design. RM appraised all papers, with SH and MW checking a different 20% each of the studies for accuracy. Any disagreement was discussed to achieve agreement.

### Data analysis

The JBI meta-aggregation approach was utilised to analyse the qualitative data to categorise, quantify and summarise the broad findings on barriers and enablers to prehabilitation [32]. Inductive content analysis was used to organise and present synthesis categories to aid in highlighting the patterns of barriers and enablers across multiple populations, surgical and cancer types and various health systems [33]. We acknowledge that frequency does not necessitate higher importance but rather, intends to identify potential widely held experiences. This was deemed more appropriate to apply to our analysis.

Theoretically, the intention was to investigate barriers and facilitators to prehabilitation from different stakeholder perspectives. However, it became clear that health system and implementation factors are significantly important and hence the analysis was expanded to include these factors.

Data analysis utilised both inductive and deductive approaches. One author (RM) read and collated key results and themes for each paper. Each study’s key findings were initially coded inductively by the same author (RM), highlighting barriers and facilitators to prehabilitation. These codes were grouped into themes and counted for frequency in the data. These themes were generated by three members of the authorship team (RM, SH and MW). Themes were discussed and combined where appropriate, and refined and confirmed further for clarity. Extracted quotes were utilised to best represent the theme. Themes were expanded beyond simplistic barriers and facilitators into the patient and implementation constructs. This method acknowledges how themes can be both barriers and facilitators when in different contexts. This method of analysis enabled deep and wide exploration of the data.

## Results

**Fig. 1:**
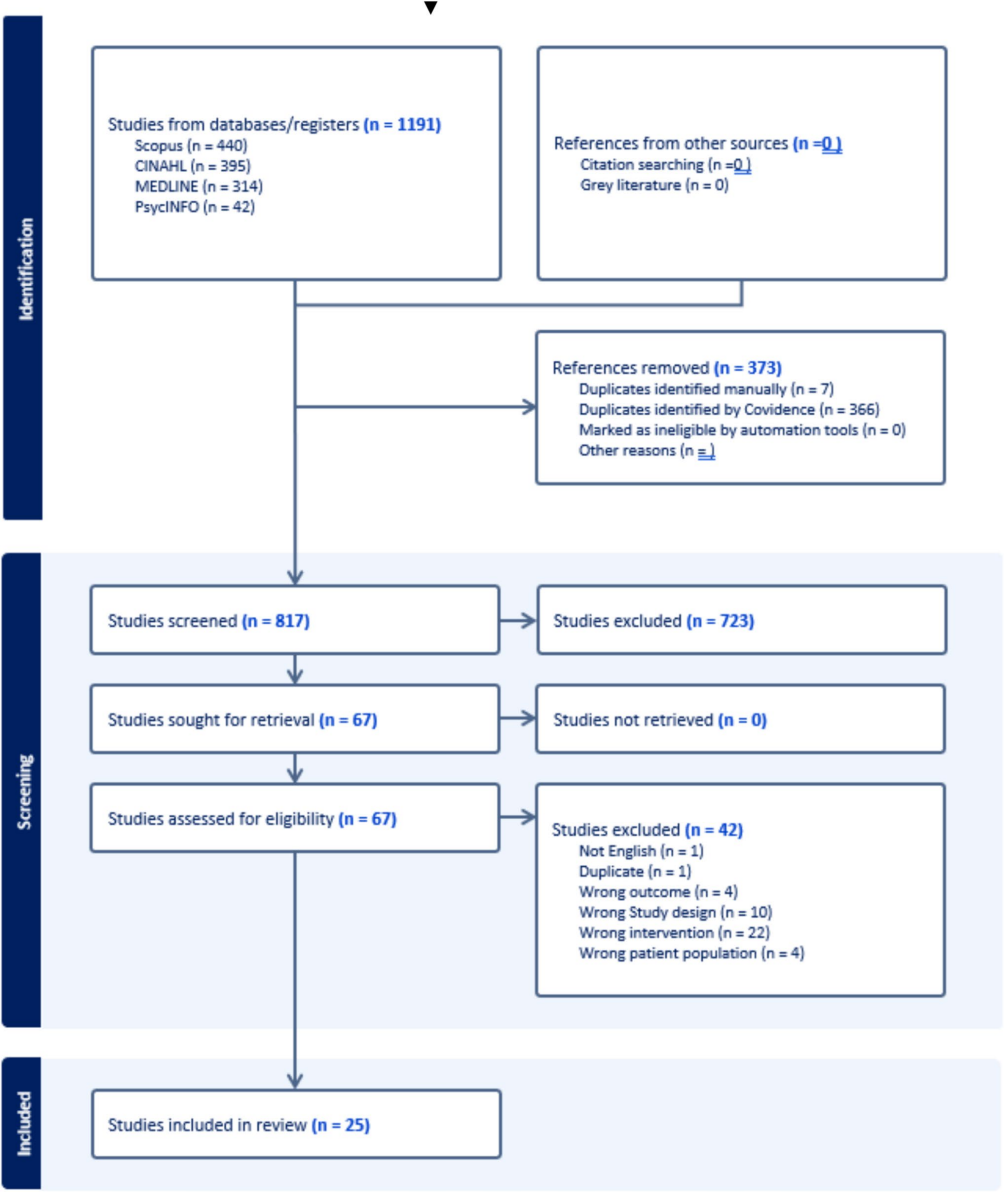
PRISMA figure

Twenty-five papers were identified and included in this review. See the PRISMA figure (Fig. 1).

### Study characteristics

Cancer types varied across the 25 included studies: varied tumour types (8/25 studies [34–41]), colorectal (5/25 studies [26, 42–45]), breast (3 studies [21, 46, 47]), lung (2 studies [24, 48]), ovarian (2 studies [49, 50]) and single studies for bladder [51], esophagogastric [52], pancreatic [53], kidney [54] and prostate cancers [55]. Twenty-one studies [21, 26, 34, 36–38, 40–49, 51–55] were conducted within research trials and four were conducted in established real-world prehabilitation programs [24, 35, 39, 50]. Ten were unimodal (namely exercise [21, 37, 43, 44, 47–49, 51–53]), 14 were multimodal (all including exercise ± nutrition or psychological intervention [24, 34–36, 38–42, 45, 46, 50, 54, 55] and one study did not report the prehabilitation intervention due to various experiences by study participants [26]. Study characteristics can be found in the supplementary files.

### Quality assessment of included studies

The quality of included studies was assessed using the Mixed Method Appraisal Tool (MMAT). Twenty-three of the 25 the papers scored 100% for the qualitative elements and two scored 80%. The table of MMAT scores is in Table 2.

**Table 2:** MMAT scores of included studies

Study and ref number (*X*)	Author	Type of study	Screening questions	Qualitative studies	Quantitative randomised controlled trials	Quantitative non-randomised studies	Quantitative descriptive studies	Mixed-methods studies	MMAT average percentage of criteria met across relevant study methods
1 [51]	Banerjee et al. (2021)	Qualitative	YY	YYYYY (100%)				-	100%
2 [34]	Barnes et al. (2023)	Qualitative	YY	YYYYY (100%)				-	100%
3 [42]	Beukers et al. (2024)	Mixed methods	YY	YYYYY (100%)			YYYNY (80%)	YYYYY (100%)	93%
4 [35]	Bingham et al. (2023)	Qualitative	YY	YYYYY (100%)				-	100%
5 [46]	Brahmbhatt et al. (2024)	Mixed methods	YY	YYYYY (100%)	YYYNY (80%)			YYYYY (100%)	93%
6 [21]	Brahmbhatt et al. (2020)	Mixed methods	YY	YYYYY (100%)			YYYNY (80%)	YYYYY (100%)	93%
7 [43]	Burke et al. (2015)	Qualitative	YY	YYYYY (100%)				-	100%
8 [47]	Casanovas-Alvarez et al. (2024)	Qualitative	YY	YYYYY (100%)				-	100%
9 [24]	Collaço et al. (2022)	Qualitative	YY	YYYYY (100%)				-	100%
10 [52]	Cooper et al (2022)	Qualitative	YY	YYYYY (100%)				-	100%
11 [36]	Drummond et al (2022)	Qualitative	YY	YNYYY (80%)			YNYNY (60%)	YYYNY (80%)	73%
12 [48]	Finley et al. (2020)	Mixed Methods	YY	YYYYY (100%)			YYYNY (80%)	YYYNY (80%)	87%
13 [26]	Heil et al. (2022)	Qualitative	YY	YYYYY (100%)				-	100%
14 [49]	Jespersen et al. (2024)	Mixed methods	YY	YYYYY (100%)			YYYYY (100%)	YYYYY (100%)	100%
15 [37]	Mao et al. (2023)	Mixed methods	YY	YCYYY (80%)			YYYNY (80%)	YYYYY (100%)	87%
16 [38]	Moyen et al. (2025)	Mixed methods	YY	YYYYY (100%)			YYYNY (80%)	YYYYY (100%)	93%
17 [44]	Murdoch et al. (2021)	Mixed methods	YY	YYYYY (100%)			YNYNY (60%)	YYYYY (100%)	87%
18 [53]	Parker et al. (2019)	Mixed methods	YY	YYYYY (100%)		YYYYY (100%)		YYYYY (100%)	100%
19 [54]	Paulo et al. (2023)	Qualitative	YY	YYYYY (100%)				-	100%
20 [55]	Pedersen et al. (2025)	Qualitative	YY	YYYYY (100%)				-	100%
21 [39]	Powell et al. (2023)	Qualitative	YY	YYYYY (100%)					100%
22 [40]	Randall et al. (2025)	Mixed methods	YY	YYYYY (100%)		YYNYY (80%)		YYYYY (100%)	93%
23 [50]	Saggu et al. (2025)	Qualitative	YY	YYYYY (100%)				-	100%
24 [45]	Sier et al. (2024)	Qualitative	YY	YYYYY (100%)				-	100%
25 [41]	Wu et al. (2022)	Qualitative	YY	YYYYY (100%)				-	100%

### Overview of results

The 25 included studies reported on multiple factors that influence engagement in prehabilitation for cancer surgery programs. Given this is a rapidly growing field, we identified included studies that focused mostly on patient engagement factors, implementation factors or a combination of both, so we present the results according to these broad themes. Within each theme, subthemes are presented. An overview of themes and subthemes is presented in Table 3.

**Table 3:** Overview of themes

Theme	Subtheme	Supporting studies
Patient engagement factors	Relationships—invitation moment, connection with clinicians Social support (pre-existing vs curated with program)	[21, 24, 26, 34–55]
	Individualised content (targeted, realistic, meaningful)	[21, 24, 26, 34, 35, 37–41, 43, 45, 47, 49–55]
	Personal circumstances (time commitment, responsibilities, symptoms, exercise experience)	[21, 24, 26, 34, 35, 37–39, 41–54]
	Belief in benefit	[21, 24, 26, 34–47, 49–52, 54, 55]
Both patient engagement AND implementation factors	Awareness/education (clinician, community)	[26, 35, 39, 44, 47]
	Delivery options (content, location, flexibility)	[21, 26, 36–38, 40, 41, 43, 45–50, 52–55]
Implementation factors	Tyranny of timing	[26, 35, 39, 44]
	Workforce capability and capacity	[21, 24, 26, 35, 39, 44, 45, 47, 50]
	Wider system support (funding, space, administration) operational resources and the built environment	[24, 26, 35, 39, 44, 47]

### Theme 1: Patient engagement factors

This theme focused on the factors that influenced engagement that are solely connected with patient engagement. We identified patient engagement factors reported in all 25 studies. We identified 4 subthemes which include motivational relationships, meaningfully individualised content, personal circumstances and the belief of benefit.

#### Motivational relationships

The relationship between the clinician and patient was a key facilitator to engagement in 23/25 studies. This includes the initial invitation or education regarding prehabilitation, being more motivational if introduced by a medical professional (9 studies [24, 39, 41, 45, 48, 50, 51, 53, 55]. Additionally, ongoing relationships with clinicians delivering prehabilitation were also described as beneficial for accountability, monitoring of progress and general support (23 studies [21, 24, 26, 34–41, 43–47, 49–55].

*“I needed the support of [my surgeon] saying, ‘This is pretty critical. The stronger you are going into surgery, the stronger you are when you’re going to recover”* [53]

*“You never feel alone when you know you’re on a little programme and you’re going to get a phone call. And you’re trying your best because if you doing something for yourself to get through what you’ve got to go through, it all helps.”* [52]

Social support of family and friends was also identified as an enabler for patients engagement in 16 studies [34–36, 38, 41, 42, 45–47, 49–55].

*“Now, [my partner] wants me to do the exercises so I’m ready for the operation and that, as opposed to if I was alone and doing them alone. Maybe I would have not done them as much as I did”* [34]

*“I was talking to my children about it and they said ‘yeah mom, it would be good for you, for your endurance’. Especially since I have problems with my lungs. Yes, they both said ‘Mom, it would only be the best for you, because you are under good guidance and then you’ll at least be a bit stronger for the surgery”* [45]

#### Personal circumstances

Uniquely personal circumstances were reported in 23/25 studies as engagement barriers and facilitators. Exercise experience (6 studies [34, 39, 45, 51, 53, 55]) and emotional responses around diagnosis (13 studies [21, 34, 35, 38, 39, 41–43, 46, 50, 52, 54, 55]) were reported as both barriers and facilitators, whereas cancer-related symptoms or comorbidities (15 studies [24, 34, 38, 41, 42, 44–50, 52–54]), family or work commitments and responsibilities (9 studies [21, 37, 41, 42, 45, 46, 48, 50, 53, 54]) and perceived lack of time (7 studies; [21, 24, 26, 35, 39, 44, 54]) were reported as barriers.

*“I was pretty much home bound in between the pain, if I didn’t take the pain medicine, I couldn’t think straight it hurt so bad and if I did take it I was just kind of out of it”* [54]

*“between work and family and different things like that, that influences your time”* [54]

*“Lack of time was another important barrier that was commonly referred to by participants because the preoperative period is typically occupied with many medical appointments and personal/professional responsibilities.”* [21]

*“I’ve always been fairly active. So, the transition wasn’t hard”* [53]

#### Individualised content

Individualised prehabilitation was reported in 22/25 studies [21, 24, 26, 34, 35, 37–41, 43, 45, 47, 49–55]. Frequently, this referred to the prehabilitation programs being manageable, achievable and realistic and tailored to the individual.

*“it were all done professionally and you were assessed before you were given exercises, they were tailor made to your individual needs […] so yeah, no concerns at all”* [39]

#### Awareness and belief in benefit

Personal belief in the benefit and importance of prehabilitation was reported as a facilitator in 23/25 studies [21, 24, 26, 34–47, 49–52, 54, 55]. This was reported in terms of the patient noticing the physical benefits themselves in their general life before the operation, but also as being critical in the context of influencing their recovery.

*“I really feel like I benefited a lot from it because it caught me in that time just after diagnosis when things were pretty scary and pretty awful and I felt like it was one of the key pieces of my plan for positivity during this whole thing, because it was setting a tone for recovery.”* [21]

*“To realize that it is a major operation, that you have to be fit for…Some patients need a wake-up call”* [26]

*“The day after, I already walked down the hallway and back, as they advised me. So, I assume that, if I hadn’t participated, it might have been harder to already be able to do that. That is possible. I can’t prove it”* [45]

### Theme 2: Implementation factors

This theme included factors that illustrate how prehabilitation is implemented or operationalised is often a significant factor for patient engagement. This theme focuses on specific implementation and service aspects that influence patient engagement to prehabilitation which was reported in 9/25 studies. This included the tyranny of preoperative timing (4 studies), workforce capability and capacity (9 studies) and operational resources and the built environment (6 studies).

#### Tyranny of pre-operative timing

The complexity of the timing of prehabilitation from an implementation perspective refers to several factors that are seen most as barriers. This included the timing of invitation in 4/25 studies [26, 35, 39, 44]; being sensitive to time of diagnosis but ideally utilising as much preoperative time effectively. The short diagnosis to surgery interval was identified as a barrier to effective implementation as well as the complex coordination of patient care through multiple clinician services during this often-stressful preoperative interval.

*“Oh, as early as possible, to be honest. I think if the patient gets a lot of information at diagnosis and sometimes it’s, you know, it’s been a big blow and they maybe aren’t able to cope with that and I think the clinical nurse specialists at that time breaking bad news appointment are so far the best positioned people in order to make a judgement call on what, how much, or how little the patient can actually deal with on that particular day. But as early as possible, any kind of rehabilitation, the earlier you can get started then that, you know, you expect better outcomes.”* [35]

*“If patients come late for decision to surgery and they have less than 2 weeks to be operated upon to avoid target breach, it its not feasible to refer them to the program”* [39]

*“Logistically it is quite complex. For example: patients have to come back to the physical therapist on Monday. But we cannot see the patient on Monday because we are scheduled to be on the operation room for the whole day.”* [26]

#### Workforce capability and capacity

This subtheme refers to clinician training and knowledge to provide prehabilitation as both a barrier and an enabler in 5/25 studies [26, 35, 44, 45, 47] and clinician capacity and availability to deliver said prehabilitation as a barrier in 9/25 studies [21, 24, 26, 35, 39, 44, 45, 47, 50].

*“the patients with experience in physiotherapy in the community preferred the in-hospital training because, in their experience, physiotherapists in the community generally have less time, attention, and expertise”* [45]

*“When you’re having very specific surgery, where someone is actually familiar with it, it’s not like I can call up a physio center and say, ‘oh can you help me because these problems.’ You need to have someone who specializes and recognizes what the issues are...”* [21]

#### Operational resources and built environment

This subtheme refers to the service workflow inclusive of referral pathways and care coordination (6/25 studies; [24, 26, 35, 39, 44, 47]), staffing logistics and physical resources and equipment (4/25 studies; [26, 35, 44, 47]). Funding for a sustainable service from implementation was also identified as a key factor in 2 studies (2/25 studies [26, 35]). Administrative support issues are inclusive of resource management (3/25 studies [26, 35, 47]), system integration and support, as well as sustaining a prehab program. This subtheme also involves the broader health system and hospital strategy such as proactive leadership, clinical ambassadors, shared vision with strategic stakeholders and support from both hospital and national levels as discussed in 2/25 studies [26, 35].

*“We have the advantage of having a specialized nurse who follows the patient throughout the process, during all appointments with different specialists… This helps to embed these kind of processes thoroughly…”* [26]

*“It would be nice if financial support is guaranteed, especially because both the dieticians and physical therapist need extra fulltime equivalents.”* [26]

*“The overarching strategy of the hospital where I work is to make the region the healthiest region in the Netherlands and everything is aimed at that. Prehabilitation fits well in this strategy and that helps a lot to get things done.”* [26]

### Theme 3: Combination factors

This theme included all the factors that more directly affect both patient engagement and implementation factors. The subthemes were awareness and education (5/25 studies; [26, 35, 39, 44, 47]) and delivery options (18/25 studies; [21, 26, 36–38, 40, 41, 43, 45–50, 52–55]).

#### Clinician awareness

Clinician awareness of prehabilitation and the potential benefits were highlighted as a critical issue for patient engagement (5/25 studies; [26, 35, 39, 44, 47]). If the concept is not known conceptually with the clinician or if the clinician is not aware of the available prehabilitation services, the patient is less likely to engage. On the other side, varying level of trust in the available evidence for prehabilitation was a barrier (1/25 study; [26]).

*“We had a patient who went to another medical specialty, not involved in prehabilitation. That person thought it was quite a tough program and said to the patient: ‘Take it easy’ and the patient followed this advice.”* [26]

*“I’m not convinced yet. While the need of prehabilitation is not currently proven, the implementation takes a lot of effort. Perhaps better selection of patients who really need it should be prioritized”* [26]

#### Delivery options—where, who and who with

How prehabilitation was actually delivered was reported as both a patient engagement and implementation factor. Delivery options were also seen as implementation factors—with many studies suggesting flexibility to enable patient engagement to remove logistical barriers of travel and parking.

Appropriate delivery mode of prehabilitation (18/25 studies) was described as an important enabler in regard to patient engagement having either in-person, hospital-based, home-based, supervised, unsupervised elements or a combination [21, 26, 36–38, 40, 41, 43, 45–50, 52–55]. Another key delivery variable was in regard to the type of professional delivering the prehabilitation service, most commonly reported as an oncology exercise professional/specialist being an enabler to participation (8/25 studies; [21, 40, 43, 45–47, 54, 55]).

Manufactured social support with group exercise or education sessions was often an enabler for patients, but this was sometimes seen as a barrier to engagement for other patients as identified in 14/25 studies [21, 24, 26, 34, 37, 41, 43–45, 47, 50–52, 54].

*“Having prehabilitation outside of the hospital setting made things easier. I wasn’t feeling good with the pain and couldn’t travel too far. Could also do it in my own time”* [41]

*“I immediately saw a group of people in the same situation as me. I mean, it was the bald group, and we were all in the same place”* [47]

*“Yes, yes it [fitness center in the hospital] was at its finest. I think it’s a good location, good devices, good therapists, also I thought the guidance was excellent.”* [45]

## Discussion

This systematic review examined 25 studies that explored patient and clinician experiences regarding prehabilitation for cancer surgery. We organised the barriers and enablers into two broad themes: patient engagement and implementation factors. Patient engagement themes included relationships with clinicians, social support, individualised content, awareness and belief in the benefit of participation and acknowledging personal circumstances. Implementation factors including workforce capability and capacity, time pressures, wider system support and operational resources are likely to influence clinicians and systems and therefore impact the ability to get prehabilitation programs into practice. There are also several factors that impact both patient and implementation domains: awareness and education of clinicians and community, and the delivery options of prehabilitation.

Earlier literature identifies numerous patient level factors—namely through behaviour change theories [34, 56–58]. However, there is a lack of implementation success solely through behaviour change strategies, evidenced through recent literature focusing more on system level factors [59–61]. As shown, only four studies investigated perspectives of embedded programs [24, 35, 39, 50]. Therefore, future research needs to consider both individual and system level elements to facilitate sustainable and effective implementation but also to utilise successful embedded programs experiences. Our review synthesised both research trials with the experiences of those in embedded programs to highlight key elements for successful implementation and to suggest directions for further investigation.

### Relationships

This review emphasises the pivotal role of the patient-clinician relationship in prehabilitation. Evidence consistently demonstrates that this relationship is central to patient engagement, particularly during the initial introduction of prehabilitation [24, 39, 45, 46, 50, 53, 55]. Emerging models that prioritise solely digital solutions, such as app-based or AI-driven platforms, aim to improve access, equity and reduce clinician workload; however, these approaches risk diminishing the vital relational aspects of care. Shifting responsibility to patients during a time-sensitive and stressful period may compromise adherence and continuity of care [62, 63]. Furthermore, it remains unclear whether such technologies can match the engagement outcomes and experience achieved through traditional clinician-led interactions. A potential solution likely lies in hybrid models that integrate digital tools with clinician oversight. These models could reduce clinical workload while preserving the relationship component critical for sustained engagement, though this approach requires further investigation.

Positive experiences reported by both patients and clinicians in prehabilitation highlight the role in fostering trust in the health system and enhancing professional satisfaction. Importantly, patients often face information overload at diagnosis and before surgery; therefore conveying benefits of prehabilitation face-to-face by key clinicians remain essential for adherence [21, 24, 37–47, 49–52, 54, 55]. Future prehabilitation strategies should build on existing evidence, maintaining the relational dimension of care while extending the invitation to family or caregivers as key facilitators [64]. Research should explore mechanisms for strengthening patient-clinician relationships within evolving service models at both the individual and system levels, given the well-established critical influence on engagement.

### Flexibility of delivery

In addition to maintaining the clinician-patient relationship as described above, patients from various research trials utilising various delivery methods frequently identified that a hybrid approach is preferable for delivering prehabilitation. The patient’s identified desire for the combination of face-to-face, virtual or phone contacts illustrates that health services need to remain constantly flexible—which is frequently difficult within research trials. Hence, future prehabilitation trials and programs should allow for patient preference on *how* they engage with prehabilitation, rather than only *if* they engage in a set delivery method. Home-based prehabilitation trials have shown effectiveness for adherence and postoperative complications in recent systematic reviews which invites more confidence with this mode of delivery [65, 66]. This is likely to be facilitated through virtual or phone interactions as evidenced by 14 of our included studies [21, 36–42, 44, 46, 49, 52, 53, 55].

Centralisation of cancer care and surgery in centres of excellence has been shown to have better healthcare outcomes [67]. However, this may create barriers to those that have further distance to travel and those with less resources to receive care at these centres [68]. Rather than expect each health system to develop and implement their own prehabilitation program, it may be wise to identify such centres of excellence to invest in workforce capability and capacity to pool resources and expertise and allow for a more seamless delivery of healthcare in or from one setting—rather than flowing between hospital and community settings. While the UK has several examples of the hospital-community settings interfacing together, it is not easily translated into other international health systems [24, 35, 50]. However, there may be an opportunity for centralised services to deliver optimal appropriately resourced care virtually—removing logistical barriers but still accessing optimal care standards from experienced services.

### Shifting responsibility from the patient

Many trials report high rates of patient refusal for prehabilitation, citing disinterest or logistical barriers. However, many qualitative studies often show strong patient interest, suggesting unreported factors such as research-related burden (extra paperwork, commitments) and perceived optional nature of trial participation. This being compounded where patients may try to “simplify” their lives and avoid seemingly optional tasks in stressful times. How clinicians and researchers present prehabilitation also influences uptake [35, 39, 44, 47, 51]. Additionally, rigid delivery formats—despite individualised content—may limit acceptability, reflecting a service design gap. Real-world or observational studies could better capture patient complexity and choice, and therefore inform more flexible delivery models. Calls for implementing prehabilitation in practice highlight the value of observational data from embedded programs, rather than waiting for “the ideal” format that may be too context-specific for broad application across diverse cultures and health systems [29].

### Considerations for future trials

Prehabilitation programs within research are often rigid and too idealistic for real-world implementation. Future trials must prioritise formats that are effective, feasible and acceptable to patients, family members, clinicians and health services. Research should shift its focus from solely on patient-related barriers, to consider the broader context that includes the clinicians and health systems that operationalise prehabilitation.

Researchers should draw on pragmatic clinical trial design and implementation frameworks to understand the recipient, the innovation and the context in which a program is set to exist and facilitate effective implementation rather than initiate a trial [69–71]. There is already an emerging movement to these designs as evidenced by published literature [72–77]. Utilising implementation frameworks early in trial design can improve uptake and sustainability, which is increasingly important in the absence of consistent or significant research funding. In support of this, a recent systematic review found observational studies showed better outcomes than the included RCTs indicating a more balanced approach may have benefit [78].

Calls for large international multisite RCTs may continue to demonstrate clinical effectiveness, however, without the due diligence of accounting for diverse health systems, prehabilitation risks remaining a research-only or unattainable ideal concept. We implore future trials to incorporate implementation frameworks and cost-effectiveness data that are vitally important to influence health system strategy and policy-level investment. As research prehabilitation programs exist at present, to ask for this requires significant commitment and trust from health services and governments without some firm implementation structure.

### Strengths and limitations

A significant strength of this review is that it is the first systematic review of qualitative studies that has included studies where participants participated in a form of prehabilitation for cancer surgery. While most studies are research trials and not from real-world embedded services, it highlights the perspectives from patients and clinicians who have grounded lived experiences of prehabilitation. We only included and synthesised qualitative data which may reduce the breadth of our recommendations, but we believe it provides a deeper understanding of crucial elements that could be leveraged to effective implementation and engagement. There are several limitations of this review. These include the exclusion of non-English studies, and the exclusion of quality improvement initiatives which may yield more context-specific information regarding implementation.

## Conclusion

Our review demonstrates several patient factors that influence patient engagement from a bottom-up perspective, as well as health system circumstances that affect prehabilitation at a higher level, and elements that are considered both patient and implementation factors. Rather than focus solely on patient factors in isolation, we encourage future prehabilitation efforts to consider the health system and the clinicians as critical factors to the successful engagement of patients and for the implementation of prehabilitation. Prehabilitation engagement should be based in the clinician-patient relationship, delivered in an individualised mode (potentially virtually by a centralised service), and implementation should be guided by existing frameworks with heavy focus placed on the context domains. This review adds new knowledge to translating prehabilitation into routine practice by making key recommendations depending on the health system’s readiness and ability to implement this innovation, rather than just focusing on patient factors.

**Table:** 

Key recommendations	
Clinical recommendations	Practice and policy recommendations
1. Prehabilitation should be embedded within supportive cancer care and introduced early with referral to maximise engagement 2. Clinician endorsement with clear consistent messaging remains critical to patient engagement 3. Flexible and patient-centred models utilising in-person and remote options to overcome frequent barriers are essential 4. Preservation of relational care is vital to ensure engagement and adherence, regardless of digital or home-based options	1. Health systems should prioritise integration of prehabilitation into routine surgical oncology pathways 2. Sustainable implementation requires investment in a skilled and specialised workforce, as well as support from administrative and organisational infrastructure 3. Future service development and research needs to emphasise pragmatic and implementation-ready models that reflect real-world clinical contexts, health system constraints and patient complexity

## Supplementary Information

Below is the link to the electronic supplementary material.

ESM1 (DOCX 53.1 KB)

## Data Availability

No datasets were generated or analysed during the current study.
