# Supplementary material for: Prehabilitation for cancer surgery: a systematic review of qualitative literature from experienced stakeholders
Source: Support Care Cancer. 2026 Jun 9;34(7):634. doi: 10.1007/s00520-026-10811-x (PMC13249678; doi:10.1007/s00520-026-10811-x)
Supplement: Supplementary file 1 — (DOCX 53.1 KB) [file 520_2026_10811_MOESM1_ESM.docx]

| **Study and ref number (X)** | **Author** | **Title** | **Type of study** | **Cancer Type** | **Context and type of prehabilitation (unimodal, multimodal, supervised, timeframe)** | **Research setting or Real World setting** |
| --- | --- | --- | --- | --- | --- | --- |
| 1(51) | Banerjee et al, 2021  Journal: Disability and Rehabilitation,   Country: USA | Patient perspectives of vigorous intensity aerobic interval exercise prehabilitation prior to radical cystectomy: a qualitative focus group study | Qualitative, n=14 patients | Bladder cancer | UNIMODAL  Twice weekly supervised aerobic vigorous intensity interval exercise.   Duration:3-6 weeks   * Reported in primary paper Banerjee 2018 (79). | Research trial |
| 2 (34) | Barnes et al 2023  Journal: BMC Geriatrics  Country: Canada | Barriers and facilitators to participation in exercise prehabilitation before cancer surgery for older adults with frailty: a qualitative study | Qualitative, n=15 patients | Colorectal, thoracic, hepatobiliary, urologic cancers | MULTIMODAL  Home-based involved aerobic, resistance exercise, and nutritional advice.  Duration: at least 3 weeks, mean of 5 weeks. | Research trial |
| 3 (42) | Beukers 2024  Journal: Supportive Care in Cancer  Country: Netherlands | Nutritional intervention during teleprehabilitation pilot study in high-risk patients with colorectal cancer: adherence, motivators and barriers | Mixed methods n=11 patients | Colorectal cancer | MULTIMODAL Teleprehabilitation - physical exercise training and nutritional modules, face to face intake, weekly phone.   DURATION: 3-4 weeks  * Reported Fransen 2022 (80) | Research trial |
| 4 (35) | Bingham 2023,  Journal: PLOS One  Country: UK | A qualitative evaluation of a multi-modal  cancer prehabilitation programme for  colorectal, head and neck and lung cancers  patients | Qualitative n=9 patients, n= 24 clinician and stakeholders, | Colorectal, lung or head and neck cancer | MULTIMODAL  3x HIIT supervised sessions per week, with additional strength and aerobic for higher risk groups, Nutritional advice general, then increasing support with higher risk. Hybrid delivery.  DURATION: not reported | Real world setting |
| 5 (46) | Brahmbhatt 2024   Journal: Annals of Surgical Oncology  Country: Canada | A Feasibility Randomized Controlled Trial of Prehabilitation  During Neoadjuvant Chemotherapy for Women with Breast  Cancer: A Mixed Methods Study | Mixed Methods: n=6 patients | Breast cancer | MULTIMODAL  Unsupervised, home-based, 4-5 session per week aerobic and resistance. Biweekly phone calls.   Dietetic support and advice,  Stress management session.  DURATION: not reported | Research trial |
| 6 (21) | Brahmbhatt et al, 2020   Journal: Frontiers in Oncology  Country: Canada | Feasibility of Prehabilitation Prior to Breast Cancer Surgery: A Mixed-Methods Study | Mixed Methods. N=5 patients, n=2 clinicians | Breast cancer | UNIMODAL  Unsupervised, home-based, 3-5 session per week aerobic and resistance. Biweekly phone calls.   DURATION: 31 days average | Research trial |
| 7 (43) | Burke et al, 2015   Journal: Psychology of Sport and Exercise  Country: UK | Exploring the experience of adhering to a prescribed pre-surgical exercise program for patients with advanced rectal cancer: a phenomenological study | Qualitative n=10 patients | Rectal cancer | UNIMODAL  Supervised, HIIT cycle program, 2x week  DURATION: 6 weeks | Research trial |
| 8 (47) | Casanovas-Alvarez 2024  Journal: Journal of Clinical Medicine  Country: Spain | Experiences of Patients with Breast Cancer Participating in a  Prehabilitation Program: A Qualitative Study | Qualitative n=16  patients | Breast cancer | UNIMODAL  Supervised, twice weekly, Nordic walking training, muscle strengthening exercises and therapeutic education   DURATION: 6-9 weeks | Research trial |
| 9 (24) | Collaço et al, 2022,  Journal: Journal of Clinical Nursing,   Country: UK | Patients' and healthcare professionals' views on a pre- and post-operative rehabilitation programme (SOLACE) for lung cancer: A qualitative study. | Qualitative n=17 patients, n= 8 clinicians HPs | Lung cancer | MULTIMODAL   Varied supervised vs unsupervised,  DURATION: not reported  *both pre and post-surgical - only excluded if clearly post-surgical. | Real world setting |
| 10 (52) | Cooper et al, 2022   Journal: BMJ Open  Country: UK | Exploring factors influencing uptake and adherence to a home-based prehabilitation physical activity and exercise intervention for patients undergoing chemotherapy before major surgery (ChemoFit): a qualitative study | Qualitative n=22 patients | Oesophagogastric cancer | UNIMODAL  Home-based unsupervised aerobic and resistance, weekly phone calls.  DURATION: 8-9 weeks + 6weeks  *reported in Chmelo 2020 (81) | Research trial |
| 11 (36) | Drummond 2022  Journal: Scientific Reports  Country: Canada | Successes and challenges of implementing teleprehabilitation for onco-surgical candidates and patients' experience: a retrospective pilot-cohort study | Qualitative n=10 patients | Varied cancer types - abdominal and thoracic (colorectal, lung, retroperineal sarcoma, oesophagogastic cancer) | MULTIMODAL  Telehealth  aerobic, strength, weekly contacts,   nutrition and psych counselling  DURATION: mean 9.5 weeks | Research trial |
| 12 (48) | Finley et al, 2020   Journal: European Journal of Cancer Care  Country:  Lebanon | A feasibility study of an unsupervised, pre-operative exercise program for adults with lung cancer | Mixed Methods n=28 patients | Lung cancer | UNIMODAL  Exercise - aerobic. Surgeon recommended/prescribed  DURATION: >2weeks - 28.5 days average | Research trial |
| 13 (26) | Heil et al, 2022  Journal: Supportive Care in Cancer  Country: Netherlands | Implementation of prehabilitation in colorectal cancer surgery: qualitative research on how to strengthen facilitators and overcome barriers | Qualitative n=13 clinicians (3 didn't have experience with prehab) | Colorectal cancer | Various prehabilitation trials | Mostly in research trials |
| 14 (49) | Jespersen 2024  Journal: Gynecologic-Oncology Reports  Country: Denmark | Development and feasibility of an exercise therapy intervention for older  women with advanced epithelial ovarian cancer referred to neoadjuvant  chemotherapy prior to possible interval debulking surgery | Mixed methods n=15 patients | Ovarian cancer | UNIMODAL  Supervised and unsupervised Home-based exercise therapy program, resistance training, aerobic, supportive counselling  DURATION: median 12 weeks | Research trial |
| 15 (37) | Mao 2023  Journal: JTCVS Open  Country: US | Implementing a virtual mind-body prehabilitation program for patients undergoing thoracic surgery: A quality improvement project | Mixed Methods n=45 patients | Thoracic cancer (lung, oesophageal, other) | UNIMODAL  Twice weekly virtual supervised mind-body exercise group, aerobic and resistance. Mindful breathing  DURATION: not reported | Research trial |
| 16 (38) | Moyen 2025  Journal: Clinical Nutrition ESPEN  Country: Canada | Feasibility of a virtual multimodal prehabilitation intervention for  patients with cancer undergoing surgery | Mixed methods approach. N=24 patients | Varied (Lung, Colorectal and other) | MULTIMODAL   home based teleprehabilitation, regular contact, aerobic and resistance, supervised and unsupervised    DURATION: 4-6 weeks | Research trial |
| 17 (44) | Murdoch et al, 2021  Journal: BMC Cancer  Country: UK | Implementing supportive exercise interventions in the colorectal cancer care pathway: a process evaluation of the PREPARE-ABC randomised controlled trial. | Mixed methods n= 28 patients, n=13 clinicians | Colorectal caner | UNIMODAL  Hospital based exercise, vs home based exercise via phone, vs standard care   DURATION: 3-4 weeks | Research trial |
| 18 (53) | Parker et al, 2019   Journal: Journal of Physical Activity and Health  Country: US | Supports and barriers to home-based physical activity during preoperative treatment of pancreatic cancer: A mixed-methods study | Mixed Methods  n=10 patients | Pancreatic cancer. | UNIMODAL  Home-based, aerobic and resistance exercise program. Phone call fortnightly.   DURATION 16 weeks mean | Research trial |
| 19 (54) | Paulo 2023  Journal: European Journal of Oncology Nursing  Country: US | Barriers and facilitators to physical activity prehabilitation in patients with kidney cancer. | Qualitative n=20 patients | Kidney cancer | MULTIMODAL  detail not reported  DURATION: not reported | Research trial |
| 20 (55) | Pedersen 2025  Journal: European Journal of Oncology Nursing  Country: Denmark | Prostate cancer patient experience of prehabilitation prior to radical prostatectomy – A hermeneutical phenomenological study | Qualitative n= 8 patients | Prostate  cancer | MULTIMODAL aerobic and resistance exercise; pelvic floor exercise; screening psych/nutrition.  Home based, app based  DURATION: 4 weeks | Research trial |
| 21 (39) | Powell 2023,   Journal: BMC Cancer  Country: UK | Acceptability of prehabilitation for cancer surgery: a multi-perspective qualitative investigation of patient and ‘clinician’ experiences | Mixed methods n= 18 patient, 24 clinicians (free-text in survey) | Colorectal, Lung, Oesophagogastric | MULTIMODAL  Either supervised or unsupervised, monitoring by Exercise specialists,   screening for psych/nutrition  DURATION: not reported. | Real world setting |
| 22 (40) | Randall 2025  Journal: Canadian Journal of Anaesthesia  Country: Canada | Starting a surgical prehabilitation program: results  from a pragmatic nonrandomized feasibility study | Mixed methods  n = 4 patients * participants not specified as cancer patients | Various cancer types | MULTIMODAL  aerobic and resistance exercise, nutrition, psychological. Weekly contact  DURATION: 36 days assessment to surgery  * excluded clearly non-cancer responses | Research trial |
| 23 (50) | Saggu 2025   Journal: BMC Women's Health  Country: UK | ‘I was eager to do anything I could to improve the situation’: a qualitative study of patients’ experiences and views of prehabilitation for ovarian cancer surgery | Qualitative n=21 patients | Ovarian cancer | MULTIMODAL  Physical activity guidance, psych and nutritional support through videos, worksheets. Referred to specialist AHPs as needed.   DURATION: Up to 3 months to surgery  *excluded patient themes/quotes when clearly reported as naive | Real world setting |
| 24 (45) | Sier et al 2024  Journal: Patient Preference and Adherence  Country: Netherlands | Participation and Compliance in a Multimodal Prehabilitation Program for Colorectal Cancer (PACE): A Qualitative Study | Qualitative n=6 patients | Colorectal cancer | MULTIMODAL  Supervised in-hospital, personalized exercise program, and nutritional intervention  DURATION: 4 weeks | Research trial |
| 25 (41) | Wu et al, 2022  Journal: Clinics and Practice  Country: UK | Understanding Patients’ Experiences and Perspectives of Tele-Prehabilitation: A Qualitative Study to Inform Service Design and Delivery | Qualitative n=22 patients | Colorectal, breast, urology, unknown. | MULTIMODAL  Community based, personalised exercise, nutrition advice, smoking and alcohol advice, counselling, tele-prehabilitation program.  DURATION: not reported | Research trial |

79. Banerjee S, Manley K, Shaw B, Lewis L, Cucato G, Mills R, et al. Vigorous intensity aerobic interval exercise in bladder cancer patients prior to radical cystectomy: a feasibility randomised controlled trial. Support Care Cancer. 2018;26(5):1515-23.

80. Franssen RFW, Bongers BC, Vogelaar FJ, Janssen-Heijnen MLG. Feasibility of a tele-prehabilitation program in high-risk patients with colon or rectal cancer undergoing elective surgery: a feasibility study. Perioperative Medicine. 2022;11(1):28.

81. Chmelo J, Phillips AW, Greystoke A, Charman SJ, Avery L, Hallsworth K, et al. A feasibility study to investigate the utility of a home-based exercise intervention during and after neo-adjuvant chemotherapy for oesophago-gastric cancer-the ChemoFit study protocol. Pilot and feasibility studies. 2020;6:50.
